# Monosodium Glutamate Intake and Risk Assessment in China Nationwide, and a Comparative Analysis Worldwide

**DOI:** 10.3390/nu15112444

**Published:** 2023-05-24

**Authors:** Hangyu Yu, Rui Wang, Yunfeng Zhao, Yan Song, Haixia Sui, Yongning Wu, Hongjian Miao, Bing Lyu

**Affiliations:** 1 China National Center for Food Safety and Risk Assessment, Beijing 100021, China; yuhangyu@cfsa.net.cn (H.Y.); zhaoyf@cfsa.net.cn (Y.Z.); songyan@cfsa.net.cn (Y.S.); suihaixia@cfsa.net.cn (H.S.); wuyongning@cfsa.net.cn (Y.W.); lvbing@cfsa.net.cn (B.L.); 2 Laboratory of Nutrition and Development, Key Laboratory of Major Diseases in Children, Ministry of Education, Beijing Pediatric Research Institute, Beijing Children’s Hospital, Capital Medical University, National Center for Children’s Health, Beijing 100045, China; 15810948239@163.com

**Keywords:** monosodium glutamate (MSG), consumption/intake survey, food safety, condiments, flavoring agents, acceptable daily intake, general population

## Abstract

The sixth Total Diet Study (TDS) of China included a countrywide study to assess the health effects of MSG (monosodium glutamate). MSG detection, consumption analysis, and risk assessment were conducted on 168 samples from seven food categories of the most typical Chinese daily diet. The highest value of MSG in the daily diet of the Chinese population was 8.63 g/kg. An MSG intake of 17.63 mg/kg bw/d for the general population of China was obtained from content measurements combined with food consumption, while the data from the apparent consumption survey alone gave 40.20 mg/kg bw/d. The apparent consumption did not consider the loss of MSG during food cooking, resulting in an overestimate. To offer a global perspective, MSG content, food category contributions, and ingestion levels across nations were summarized and thoroughly investigated. A realistic, logical, and precise risk assessment protocol for MSG daily intake was developed in this article.

## 1. Introduction

Monosodium glutamate (MSG), a flavor enhancer, is typically utilized in food processing in homes and the catering sector worldwide. The dangers of excessive MSG consumption on human health have drawn researchers’ attention repeatedly [1,2,3,4,5,6,7,8,9,10,11,12]. The latest findings suggest that excessive MSG can trigger obesity [6,13,14,15,16,17], reproductive diseases [18], and neurological symptoms [19,20,21,22,23]. Although the inclusion of glutamates is often permitted up to a maximum quantity of 10 g/kg of food, as stated by European Union (EU) Regulation No. (2010/257), the controversy regarding MSG’s impact on health issues in humans has resulted in a bad public perception of the additive [24,25,26].

Hence, the European Food Safety Authority (EFSA) re-evaluated the safety of glutamic acid and its derived glutamates in June 2017 and presented a population-acceptable daily intake (ADI) of 30 mg/kg bw/d, expressed as all inorganic salt forms of glutamic acid [27]. According to the reassessment’s findings, MSG intake in European nations ranged from 8 to 198 mg/kg bw/d, which indicates that MSG intake over ADI in some European nations might be of concern. In contrast, China, the world’s largest producer and consumer of MSG, currently lacks studies and risk assessments on the health effects of MSG consumption in the general population. In the past, information on MSG intake in China was primarily gleaned from the China Health and Nutrition Survey (CHNS), which ran from 1991 to 2006 [28]. The data were obtained through a household survey of the weight difference of MSG containers in a home for three consecutive days, which was limited in tracking MSG losses in home-cooked food, and the outcomes were subject to MSG intake overestimation. Many relevant studies worldwide also adopted a similar survey approach to the CHNS to assess the population intake of MSG, which was an efficient and economical strategy [29,30,31,32,33,34]. However, with the release of the results of the EFSA reassessment, accurate human intake and risk assessment data should be upgraded. This has importance for China and the world at large in terms of human health effects, risk assessment outcomes, and even international commerce.

In this study, we investigated the types of daily diet and consumption in the general population of Chinese residents to obtain more exact information on the actual intake of MSG in the daily diet based on the sixth Total Diet Study (TDS) of China. The MSG content of all consumed foods in the ready-to-eat state was detected by our laboratory UPLC-MS/MS assay. The MSG daily intake was calculated by multiplying the food consumption and the related content. The daily intake of MSG in the general population of China was assessed at provincial and national levels based on an ADI value of 30 mg/kg bw/d for a standard Chinese man’s bw of 63 kg, on the risk of human health effects. Meanwhile, to compare the variability of risk assessment results between apparent MSG consumption and actual intake, a 3-day, 24-h dietary survey was conducted using a CHNS-like survey of over 10,000 households in a selected area covering 70% of China’s land area and 85% of its population and assessed in terms of apparent consumption. The real daily dietary intake of MSG in the general Chinese population was substantially lower than the apparent consumption, as shown by the comparison between the findings of the intake analysis and the apparent consumption analysis. This finding suggests that to appropriately assess the risk of MSG intake and prevent overestimation, losses of MSG during cooking and trans-oral processes should be taken into consideration.

## 2. Materials and Methods

### 2.1. Food Consumption Survey and Sampling

TDS, a continuous national study, monitors the levels of trace elements and harmful compounds in foods and is also used to estimate hazards of dietary exposure in Chinese populations. The China National Center for Food Safety and Risk Assessment (CFSA) conducted the sixth China TDS from 2016 to 2021 in twenty-four provinces, namely, Heilongjiang, Liaoning, Jilin, Hebei, Beijing, Henan, Shanxi, Shaanxi, Ningxia, Neimenggu, Qinghai, Gansu, Jiangxi, Fujian, Shanghai, Jiangsu, Zhejiang, Hubei, Sichuan, Guangxi, Hunan, Shandong, Guizhou, and Guangdong. In the sampling setting, provinces with a population above fifty million randomly set two different urban sampling points and four rural sampling points. Conversely, one urban sampling site and two rural sampling sites were randomly selected in provinces with a population of fifty million or fewer. The number of participants in each sampling site was not less than 30 households. According to the mixed food sample method used in the earlier TDS in China, the surveyed per capita food consumption was divided into thirteen categories, namely, cereals, legumes, potatoes, meats, eggs, aquatic foods, milk, vegetables, fruits, sugar, beverages, alcohol, and condiments (including cooking oil). The dietary composition of the surveyed areas or populations was collected by grouping the different food items consumed into the categories to which they belonged. Various food samples were collected from food purchasing points such as vegetable farms, side stores, grain stores, farmers’ markets, or farmers’ homes, near the neighborhood committee or village where each survey site was located. Food and condiments for cooking were prepared in designated restaurants, kitchens, or laboratories according to the cooking utensils and methods customarily used locally. All seasonings were added to other food groups as required by the recipe during the cooking process. Then, the ready-to-eat dietary samples were stored at −20 °C and shipped to CFSA.

### 2.2. Sample Preparation and Analysis

One hundred and sixty-eight samples from twenty-four provinces of seven food items, namely, cereals, legumes, potatoes, meats, eggs, aquatic foods, and vegetables, were examined for MSG content determination. Following the local food recipe, these foods used MSG or other analogous flavors during the cooking procedure. MSG was extracted using 4 mL of 0.1 M HCl from 1.0 g of the dietary sample thawed at room temperature during the sample pretreatments. The extraction solution was centrifuged above 4000 rpm for 10 min. Before the sample was cleaned, 1 mL of supernatant was aliquoted from the extraction solution and neutralized with 1 mL of 0.1 M NaOH. An ion exchange cartridge (MAX cartridge; 3 mL; 60 mg; Waters, Milford, MA, USA) was used for sample clean-up. Firstly, 2 mL of 5% NH_3_·H_2_O was added for cartridge equilibration. After sample loading, water (4 × 0.5 mL) and methanol (2 × 0.5 mL) were used to remove impurities. Then, 0.5 mL of 2% formic acid (*v*/*v*) in methanol was used to elute the target. All eluent fractions were combined and dried by nitrogen flow, and the residue was reconstituted in 0.25 mL of 50% aqueous acetonitrile. Finally, depending on the level of MSG content in the dietary sample, 1 mL of the eluate was diluted 10^−3^ times and then detected for content. MSG identification and quantification were performed using Shimadzu LC 8050 UPLC-MS/MS (Shimadzu, Kyoto, Japan). The positive ion mode was used in the ESI ionization. The ion spray voltage was +4.5 kV. Nitrogen was used as the atomization gas and the drying gas, with a flow rate of 3 L min^−1^ and 10 L min^−1^, respectively. The heating gas velocity was 10 L min^−1^ of air. Additionally, meanwhile, the desolvation line and heating module temperatures were 300 °C and 400 °C, respectively.

An ACQUITY^™^ UPLC HSS PFP column (2.1 mm i.d. × 100 mm, 1.8 μm) was used for MSG chromatographic separation. The temperature of the column oven was 40 °C. The mobile phase was water (phase A) and acetonitrile (phase B) containing 0.2% formic acid (*v*/*v*). The steps of the gradient elution program were: 0–3 min: 50% phase B and 50% phase A, 3–6 min: phase B content increasing linearly to 100% and kept for 2 min. The flow rate was 0.20 mL/min, and the injection volume was 1 μL. The retention time of MSG was 2.748 min under these conditions.

Quantitative and qualitative ion pairs on the multiple reaction monitor (MRM) scanning modes were 147.90^+^ > 84.05^+^ and 147.90^+^ > 56.05^+^, with collision energies of 16 eV and 23 eV, respectively, in MS separation.

### 2.3. Estimation of Dietary Intake and Risk Assessment

The dietary intake of each food category was calculated by multiplying the MSG contents and their consumption. The whole MSG intake was the accumulated sum of every food intake. Additionally, then, the sum was divided by 63 kg of the standard Chinese man for MSG intake risk assessment. The items with “not detected” data represented half of the Limits of Detection (LODs) when processing the dietary intake. The risk assessment was based on whether the general population’s intake in each province was above 30 mg/kg bw/d, and areas where MSG intake exceeds this value need to be a cause for concern.

## 3. Results

### 3.1. Validation of the Determination of Methodological Parameters

The parameters of the assay, such as LODs, Limits of Quantitation (LOQs), and spiked recovery experiment, were jointly validated by three other national laboratories. All methodological sensitivities, reproducibility, and specificities are reflected in the additional information. The LODs of MSG in the food composites ranged from 1.5 to 3.5 μg/kg in different food matrices. Additionally, the LOQs of MSG were 5.0 μg/kg in cereals, legumes, and potato products and 10 μg/kg in meat, egg, aquatic, and vegetable products, respectively. In the recovery test, blank samples were selected from the seven food matrixes in which the MSG concentrations were below the LODs. Three spiking levels of 10, 100, and 1000 μg/kg were evaluated. The validation results of this determination method are seen in Table 1.

### 3.2. Occurrence of MSG in Chinese Dietary Foods

**Figure 1:**
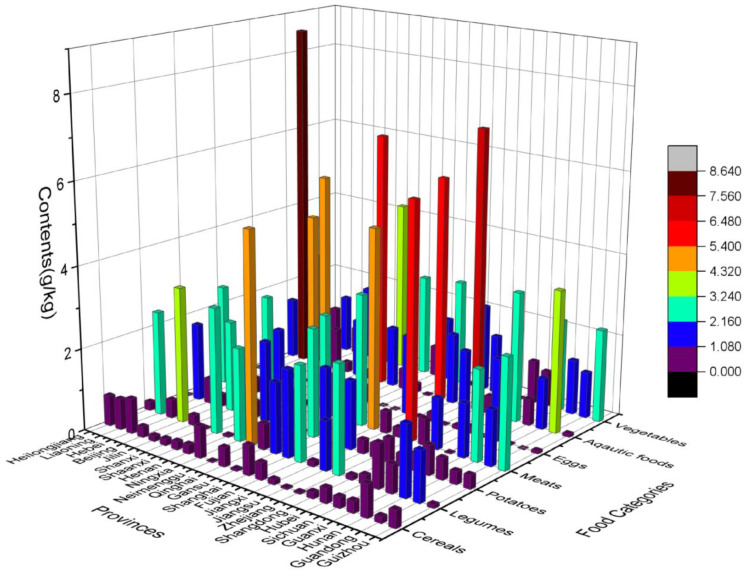
MSG levels in seven dietary groups and national mean levels in twenty-four Chinese provinces.

**Figure 2:**
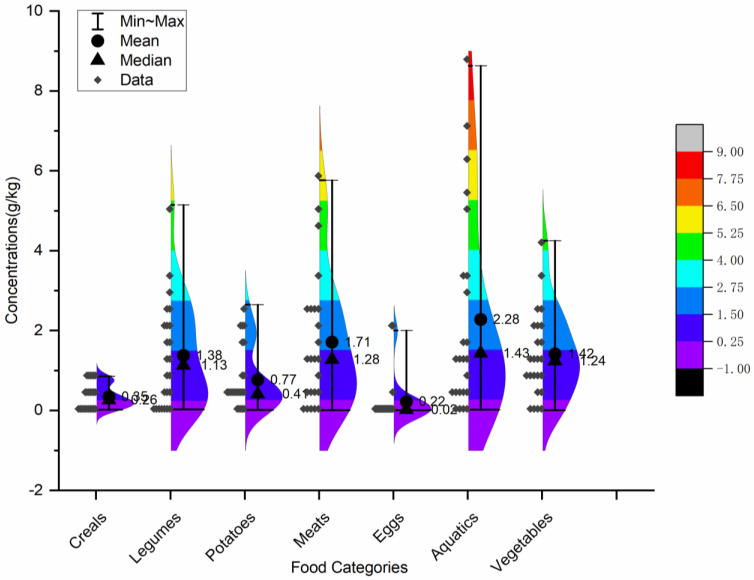
The statistics and distribution of MSG in the seven food categories.

It is clear from Figure 1 that MSG content ranged from “not detected” to 8.63 g/kg in this test. The highest amounts of cereals, legumes, potatoes, meats, eggs, aquatic foods, and vegetables were 0.85, 5.14, 2.65, 5.77, 2.00, 8.63, and 4.25 g/kg, respectively. The highest values above appeared in Hebei, Neimenggu, Gansu, Zhejiang, Guangxi, Liaoning, and Henan. As Figure 2 shows, the mean (±SD) was 0.35 ± 0.28, 1.38 ± 1.33, 0.77 ± 0.79, 1.71 ± 1.64, 0.22 ± 0.55, 2.28 ± 2.47, and 1.42 ± 1.11 g/kg in cereals, legumes, potatoes, meats, eggs, aquatic foods, and vegetables, respectively.

### 3.3. Analysis of Dietary Intake of MSG

**Figure 3:**
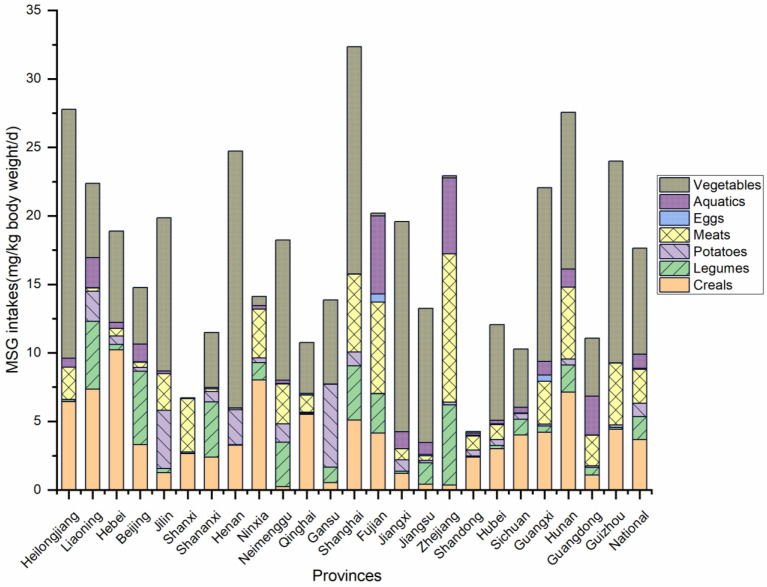
Single food category intakes, total intakes, and national mean intakes of MSG from 24 provinces and cities in China.

There was a wide range of MSG intakes, from 4.27 to 32.35 mg/kg bw/d, among different regions in China. The mean (±SD) was 17.63 ± 2.50 mg/kg bw/d at the national level. The top three highest intake provinces were Shanghai, Heilongjiang, and Hunan, with 32.35, 27.78, and 27.56 mg/kg bw/d. The province with the lowest intake was Shandong Province, with a value of only 4.27 mg/kg bw/d, as Figure 3 shows.

### 3.4. Dietary Contribution

**Figure 4:**
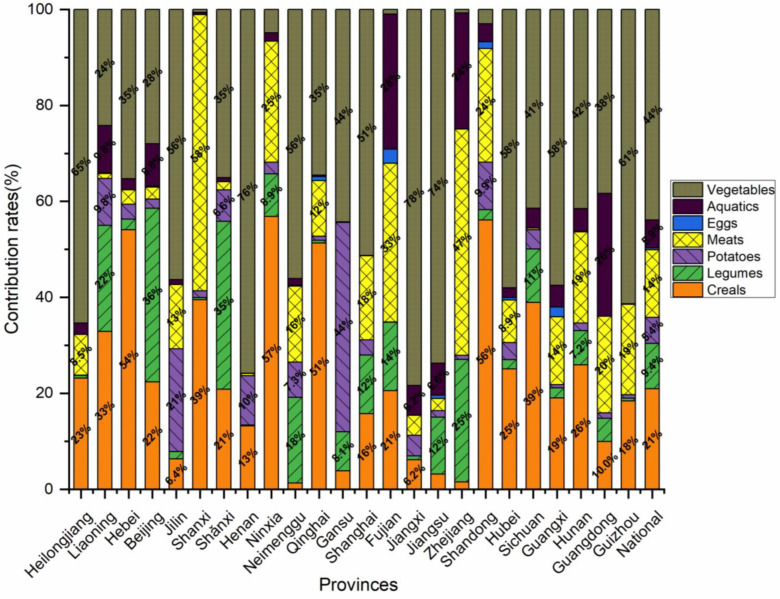
The contribution rates of MSG dietary intakes from the seven food categories from twenty-four provinces in China.

The risk evaluation included an investigation into the dietary contribution of MSG consumption. This data analysis enabled us to comprehend the variations in dietary practices between regions and to lower risk by limiting items with a high proportion of contaminants. Vegetables were the primary source of essential intake at the national level in our contribution margin analysis, contributing at a rate of 44%, as shown in Figure 4. Cereals, meats, legumes, aquatics, and potatoes were the other food groups with intake contributions above 5%. According to the contributions, they were 21%, 14%, 9%, 6%, and 5%. Among these foods, eggs had the lowest contribution rate, with a contribution of under 1%. Geographically, vegetables continue to make up the majority of each province’s MSG dietary contribution, although in Hebei, Shandong, Shanxi, Ningxia, and Qinghai, cereals take the lead. This is in line with the traditional regions of northern China, where pasta is consumed.

### 3.5. Risk Assessment

**Figure 5:**
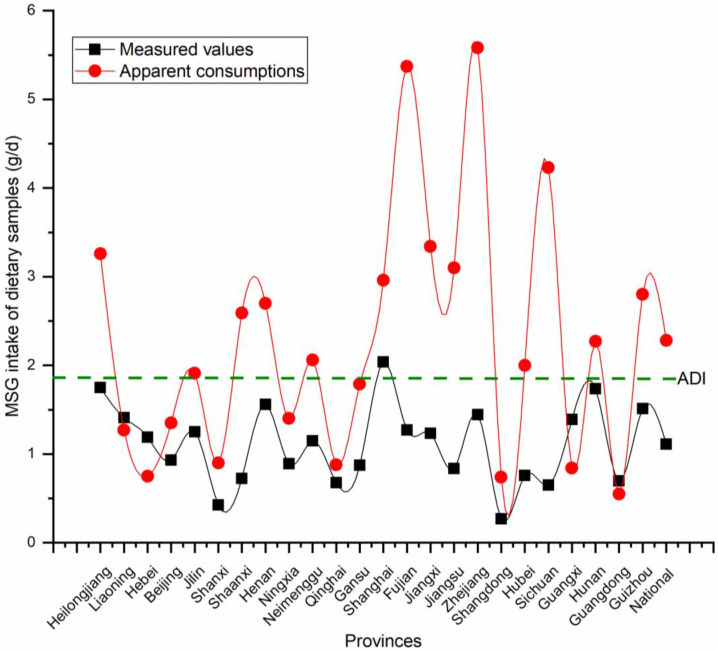
Differential results of the risk assessment between measured and apparent MSG intake values in the Chinese general population of 24 provinces in China.

Since MSG was first used in flavoring, there have been different opinions on its safety in various countries and institutions. The US Food and Drug Administration (FDA) considers the addition of MSG to foods to be “generally recognized as safe” (GRAS). In some countries and regions, MSG is even seen as the main alternative to reduce sodium salt intake. Although our group has reservations about the suitability of this ADI for the Chinese population, it has been used as a tentative evaluation criterion for the risk assessment of MSG. A uniform unit of measurement was needed for MSG intake to be consistent with the criteria used to determine results when comparing different regions of the world. The MSG intake units mentioned previously in Section 3.3 of this paper were expressed in mg/kg bw d. However, in terms of risk assessment, the standard human body weights in China and Europe were different, which introduced uncertainty in the comparison of results. For consistency in determining criteria when comparing results with different parts of the world, we multiplied the MSG intake measurements by 63 kg and divided them by 1000 to obtain the MSG intake in g/d, which eliminates the variability between different country regions. Therefore, a daily intake of MSG of less than 1.89 g/d was considered safe for Chinese people according to the ADI. As seen in Figure 5, the green dotted line represents the ADI value. The mean daily intake of MSG for the general Chinese population was 1.11 g/d. Of the 24 provinces in China, Shanghai was the only one that exceeded the green dotted line. It could be said that daily exposure to MSG in the general population in China is not a health risk. However, the results were vastly different when risk assessment has been carried out with MSG consumption survey data. The MSG apparent consumption was obtained from a 3 d, 24 h household survey, and this value was derived from the difference in the weight reduction of the MSG containers in each participating family within one day. The trend line shows that the apparent consumption and measured values in most provinces are consistent. It was worth noting that the MSG intake risk assessment resulted in 14 of the 24 provinces participating in the TDS apparent consumption survey. The top three provinces were Zhejiang, Fujian, and Sichuan, with values of 5.58, 5.37, and 4.23 g/d. The average cumulative MSG intake was 2.28 g/d. This means that the intake of the general population in China exceeds the risk threshold.

## 4. Discussion

In this study assessment, animal-based foods such as meats and aquatic had higher MSG levels than plant-based foods. Compared to plant foods, animal-based foods have more protein amino acid groups, and these free proteins may undergo hydrolysis to produce glutamate under complex external conditions. Many high-protein animal foods have high levels of MSG, and a large proportion of this should be contributed by these free glutamates [35]. This finding was helpful in our understanding of the higher MSG content in animal-based foods than in plant foods.

We also carried out a comparative analysis of the global MSG food content. As can be seen in Table 2, some long shelf-life or other ready-to-eat products such as taste cubes, sauces and soups, bovine stock, smoked bacon soup cubes, and bouillon cubes may also lead to excessive concentrations of MSG because of the flavor intent of the addition [35,36,37,38,39]. MSG content in the Chinese daily diet should not cause undue concern compared to these reports. On the other hand, the risk from catering food or commercial food intake should be noted considering the recently increasing consumption of such foods in China.

**Figure 6:**
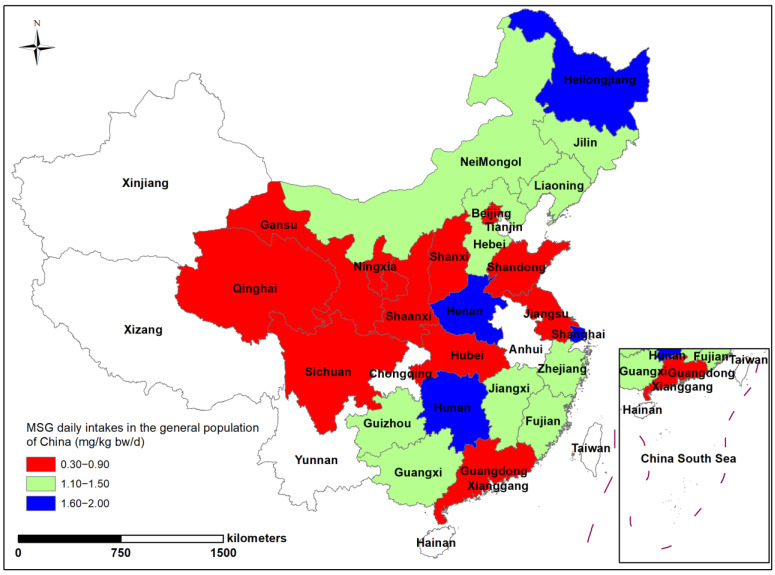
Geographical distribution of daily MSG intake levels in China.

**Figure 7:**
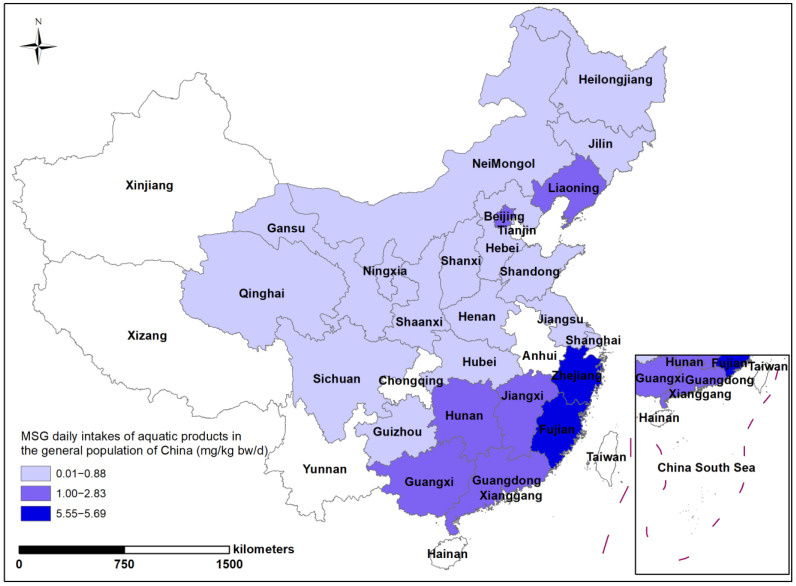
Geographical variation of MSG daily intakes for aquatic products in 24 provinces in China.

To further explore the influence of the geographical distribution and dietary consumption habits of the Chinese general population on MSG intake, we mapped the geographical distribution of total dietary MSG in China (Figure 6). In general, there was no significant regional variation in MSG intake in the total diet of the Chinese population. The highest intakes were found in Shanghai, in the Yangtze River Delta, Heilongjiang, in northeastern China, and Hunan, in south-central China. The overall intake in the western inland region was lower, at 4.30–14.80 mg/kg bw/d. However, when analyzed in terms of individual food groups, aquatics showed a clear north–south variation in the geography of intake levels, with the south generally being higher than the north (Figure 7). This was consistent with higher aquatics consumption in China’s southern provinces, as compared to its northern regions. With MSG levels as high as 8.63 g/kg, Liaoning in the north is a particular instance because intake levels there are much greater than in the northern provinces, despite aquatic consumption being at a lower level than in other provinces.

In addition, we conducted inter-country comparisons of MSG-related or similar data on the MSG dietary intake of the world population, and the results are shown in Table 3. In this study, the intake of the general Chinese population by an accurate measurement of MSG was 1.11 g/d, while the data obtained by apparent consumption alone showed 2.28 g/d, which was consistent with the data from previous CHNS surveys in China [28]. This result indicates that the loss of MSG from the actual initial addition to the final oral intake is at least 50% during daily dietary processing in China. Worldwide, the actual level of MSG dietary intake of the Chinese general population was in the middle of the range. If calculated based on apparent consumption, it was second only to the current maximum (4.0 g/d) in Thailand. The two main differences between China and Europe and the USA were how the content levels were obtained. In China, MSG levels were tested by mixing all samples according to the TDS food clustering principles and then making a mixed sample of hundreds of food items from each province. In contrast, in Europe and the USA, they were obtained from existing databases, literature reports, or from relevant companies. China excels at accurately and effectively estimating risk across a large population and geographic area. The USA and Europe are strong because they employ big data to precisely segment populations and provide more individualized solutions.

Comparatively, the European diet was dominated by high-quality baked goods, soups, broths, sauces, meat, meat products, seasonings, and condiments, all of which were high in MSG and consumed in significant amounts [29,35]. The top three sources of free glutamate intake among American adults were mostly vegetables (14.9%), protein meals (13.9%), and condiments and sauces (7.8%) [9]. This has some similarities to the structure of the Chinese diet.

In this study, we considered the intake derived by multiplying the food content and the corresponding consumption as the risk assessment value to be accurate. This strategy takes full account of factors such as the low levels of MSG added to fresh or home-cooked foods, and the fact that MSG is dissolved in the water of the dish during cooking and consumption of the food, and this water reduces the imported intake of MSG as a non-edible fraction in the actual intake. Therefore, a realistic approach to measuring MSG intake has the advantage of authenticity and precision. Although a risk assessment of health effects based on MSG consumption would lead to overestimation, high MSG consumption should be a concern in China.

In summary, our group conducted a comparison of apparent MSG consumption surveys and real intake using the TDS study to analyze the risk of MSG quickly and accurately for the public using vast data covering over 70% of China’s land and 85% of its population. This is the most representative and up-to-date MSG intake and risk assessment for China to date. We have, for the first time, examined the geographic variation of MSG intake for different food categories in China. This analysis will serve as a local point of reference and offer information on how to restructure diets healthily. However, in this paper, the results of our risk assessment were carried out mainly based on MSG intake in the general population. Therefore, we have shortcomings in the analysis of individual consumption and risk of MSG. In a subsequent study, we will reinforce the probabilistic assessment of the risk of MSG intake in the Chinese population on specific indicators such as gender, age group, and BMI for the individuals participating in the survey, to more precisely target the population groups at high risk of MSG intake.

## 5. Conclusions

In summary, we conducted a comparison of apparent MSG consumption surveys and real intake using TDS study to analyze the risk of MSG quickly and accurately for the public using vast data covering over 70% of China’s land and 85% of its population. A risk analysis of MSG intake in the general population of China was obtained from both assessments. This is the most representative and up-to-date MSG intake and risk assessment study for China to date. Additionally, considering the MSG loss caused by MSG in whole food cooking and non-edible parts of the dishes, we believe that the strategy of obtaining the maximum intake with MSG apparent consumption through a 3 d, 24 h room survey causes overestimation. This provides a more reliable technical solution for the future risk assessment of relevant condiments. We have, for the first time, examined the geographic variation of MSG intakes for different food categories in China. This analysis will serve as a local guideline and offer information on how to restructure diets healthily. Last but not least, a global comparative analysis of food content and population intake in dominant studies around the world was conducted. This provides a global view of MSG population intake and risk assessment for our colleagues around the world, providing a strategy for a rapid, accurate, and realistic MSG risk assessment of daily household dietary intakes for related work.

## Figures and Tables

**Table 1:** The method of MSG in seven food categories of TDS of LODs, LOQs, and spiking recoveries.

Food Matrix	LODs (μg/kg)	LOQs (μg/kg)	Recoveries (*n* = 6)											
			Theoretical (μg/kg)	Measured (μg/kg)	Recoveries (%)	RSD%	Theoretical (μg/kg)	Measured (μg/kg)	Recoveries (%)	RSD %	Theoretical (μg/kg)	Measured (μg/kg)	Recoveries (%)	RSD %
Cereals	1.5	5	10	9.6	96.0	2.3	100	103.5	103.5	3.7	1000	1009.8	101.0	5.6
Legume	1.5	5	10	10.2	102.0	4.1	100	112.2	112.2	2.2	1000	1024.2	102.4	4.7
Potatoes	1.5	5	10	9.8	98.0	3.4	100	115.7	115.7	6.4	1000	1120.3	112.0	6.5
Meats	3.5	10	10	7.9	79.0	4.7	100	80.1	80.1	9.2	1000	956.3	95.6	12.5
Eggs	3.5	10	10	8.2	82.0	3.2	100	107.8	107.8	5.5	1000	1120.2	112.0	2.2
Aquatic	3.5	10	10	9.2	92.0	5.4	100	101.0	101.0	6.1	1000	1104.3	110.0	9.8
Vegetable	3.5	10	10	7.7	77.0	6.0	100	122.1	122.1	8.9	1000	1190.2	119.0	14.3

**Table 2:** The summary of amounts of MSG in varieties of food matrixes in the world.

Literature	Countries	Food Matrixes and the Concentrations
Skurray G.R. et al. (1988) [38]	Austria	Fresh foods (0.077−7.57 g kg^−1^) ^a^; processed foods (0.0006 to 78.51 g kg^−1^) ^a^
Rhodes J. et al. (1991) [29]	United Kingdom	Meat and meat products (0.03–0.81%) ^b^; fish (0.39%) ^b^; vegetables (0.14–2.68%) ^b^; fruits (0.48%) ^b^; cereals (0.17–0.29%) ^b^; pizza (0.92–2.06%) ^b^; miscellaneous (0.33–8.70%) ^b^
Bodor R. et al. (2001) [39]	Slovakia	Beef stock (148.8 g kg^−1^) ^c^; meat stock (71 g kg^−1^) ^c^; vegetable soup (52.4 g kg^−1^) ^c^
Populin T. et al. (2007) [35]	Italy	Broths, soups, sauces, and salad dressings (1.29 g kg ^1^) ^d^
Acebal C.C. et al. (2008) [36]	Argentina	Beef stocks, chicken stock, stew stock (2.63 ± 0.09–11.93 ± 0.65 g dm ^−3^) a
Isa I. et al. (2009) [40]	Malaysia	Seasoning (4.8 ± 0.2–21.3 ± 0.4%) ^a^; chicken soup (5.8 ± 0.2%) ^c^; Chinese soup (7.8 ± 0.3%) ^c^; mushroom soup (2.8 ± 0.2%) ^c^
Krishna V.N. (2010) [41]	India	Masala (49.66 ± 1.34 g kg^−1^) ^c^; soup (24.59 ± 1.47 g kg^−1^) ^c^; cubes (133.50 ± 0.84 g kg^−1^) ^c^
Acebal C.C. et al. (2010) [42]	Argentina	Dehydrated meat broths, dehydrated vegetable broths (0.17 ± 0.01–1.65 ± 0.02 g dm^−3^) ^a^
Croitoru M. et al. (2010) [43]	Romania	Soup cubes, salamis, hams, vegetable mixes (0.37–119.95 g kg^−1^) ^a^
Afraa A. et al. (2013) [44]	Syrian	Cream of mushroom soup, vegetable soups, lentil soups, noodle soups, hamburgers (0.93–4.9 g kg^−1^) ^a^
Cebi N. et al. (2018) [11]	Turkey	Chips, taste cubes, sauces, soups, etc. (0.1–153.9 g kg^−1^) ^a^
This study (2023)	China	Cereals (0.35 ± 0.28 g kg^−1^), legumes (1.38 ± 1.33 g kg^−1^), potatoes (0.77 ± 0.79 g kg^−1^), meats (1.71 ± 1.64 g kg^−1^), eggs (0.22 ± 0.55 g kg^−1^), aquatic foods (2.28 ± 2.47 g kg^−1^), and vegetables (1.42 ± 1.11 g kg^−1^) ^e^

^a^: (min–max) ^b^: (means) ^c^: (*n* = 1) ^d^: (max) ^e^: (mean ± SD).

**Table 3:** Comparison of worldwide research on the intake analysis and risk assessment of MSG.

Literature	Countries	Study Type	Number of Volunteers	Protocol	MSG Intake (g/d)
Mortensen A. et al. [27]	Austria	ASNS_Adults	Volunteers comprise an unknown number of people of 6 age groups from 19 European countries.	Dietary exposures were estimated by combining individual food consumption data and maximum permitted levels (MPLs) from the EFSA European Integrated Food Consumption Database. ADI values were obtained from rodent experiments previously, and the daily MSG intake for humans was calculated at 70 kg per kg body weight.	1.41 ^a^
	Belgium	Diet_National (2004)			1.00 ^a^
	Czech Republic	SISP (2004)			1.09 ^a^
	Denmark	DANSDA (2005-08)			0.35 ^a^
	Finland	FINDIET (2012)			0.97 ^a^
	France	INCA2			0.98 ^a^
	Germany	National_Nutrition_Survey_II			0.94 ^a^
	Hungary	National_Repr_Surv			0.39 ^a^
	Ireland	NANS (2012)			0.99 ^a^
	Italy	INRAN_SCAI (2005_06)			0.54 ^a^
	Latvia	EFSA_TEST			1.23 ^a^
	Netherlands	VCPBasis_AVL (2007_2010)			1.05 ^a^
	Romania	Dieta_Pilot_Adults			0.52 ^a^
	Spain	AESAN			0.63 ^a^
	Sweden	Riksmaten (2010)			1.10 ^a^
	United Kingdom	NDNS–RollingProgrammeYears 1–3			0.99 ^a^
Rhodes J. et al. [29]	United Kingdom	National Food Survey (1987)	School children (aged 10–15) Young adults (aged 15–25)	MSG intake was obtained by measuring food content in combination with consumption.	0.58 ^b^
Sugimoto M. et al. [9]	USA	The National Health and Nutrition Examination Survey (2009–2014)	8597 children (aged 2–19) and 13,969 adults (age ≥ 20)	Food items, population information, and dietary intake of free glutamate were obtained from various national databases	0.32 ^c^
Insawang T. et al. [32]	Thailand	Epidemiological survey (2009–2010)	349 Thai adults (aged 33–55)	Giving MSG as the only source for meal preparation for 10 days.	4.00 ^d^
Thu Hien V.T. et al. [24]	Vietnam	Cross-sectional survey (2008)	1528 Vietnameseadults (age ≥ 20)	Dietary intake was obtained by the 3 d, 24 h recall survey. The consumption of MSG was assessed by weighing it for 3 consecutive days.	2.20 ^d^
Lee E.H. et al. [45]	Korea	MSG intake survey (1986)	984 Korean (age ≥ 20)	A 2 d orientation course on MSG supervision was conducted for MSG consumption. Body weight was calculated from the “4th Revised Nutrition Investigation by Koreans”.	1.57 ^d^
He et al. [28]	China	China Health and Nutrition Survey, a prospective open-cohort study (1991–2006)	10,095 healthyChinese adults (aged 18–65)	Dietary intake was obtained with a 3 d, 24 h recall survey. The consumption of MSG was assessed by weighing it for 3 consecutive days.	2.20 ^b^
He et al. [34]	China	Cross-sectional study INTERMAP (1997)	752 healthy Chineseadults (aged 40–59)	24 h recall	0.33 ^d^
This study	China	The sixth China Total Diet Study (2016–2019)	About 30,000 (aged 1–96)	See the Materials and Methods section of this article.	1.11/2.28 ^b^

^a^: This value is the mean of the MSG daily intake of adults (age: 18–64). The data were derived from the non-brand-loyal scenario section of Appendix F of EFSA’s MSG re-evaluation report [27]. ^b^: Mean MSG intake for the general population. ^c^: Mean free glutamate intake for adults. ^d^: Average MSG intake for the total population.

## Data Availability

The raw data supporting the conclusions of this article will be made available by the authors without undue reservation.
